# Alterations in Food Group Intakes and Subsequent Weight Changes in Adults: Tehran Lipid and Glucose Study

**DOI:** 10.5812/ijem.17236

**Published:** 2014-07-01

**Authors:** Firozeh Hosseini Esfahani, Hanieh Sadat Ejtahed, Parvin Mirmiran, Hossein Delshad, Fereidoun Azizi

**Affiliations:** 1 Obesity Research Center, Nutrition and Endocrine Research Center, Research Institute for Endocrine Sciences, Shahid Beheshti University of Medical Sciences, Tehran, IR Iran; 2 Department of Clinical Nutrition and Dietetic, Faculty of Nutrition Sciences and Food Technology, National Nutrition and Food Technology Research Institute, Shahid Beheshti University of Medical Sciences, Tehran, IR Iran; 3 Obesity Research Center, Research Institute for Endocrine Sciences, Shahid Beheshti University of Medical Sciences, Tehran, IR Iran; 4 Endocrine Research Center, Research Institute for Endocrine Sciences, Shahid Beheshti University of Medical Sciences, Tehran, IR Iran

**Keywords:** Body Weight Changes, Longitudinal Studies, Lipids, Glucose

## Abstract

**Background:**

The extent of weight change is varied for specific foods. This highlights the effect of dietary quality and food choices on weight control.

**Objectives:**

The aim of this study was to examine the association between alterations in food group intake and weight change over a 3 years follow-up period.

**Materials and Methods:**

This longitudinal study was conducted on 851 adults in the framework of Tehran Lipid and Glucose Study. Intakes of various foods were measured at baseline and after 3 years using a validated semi-quantitative food frequency questionnaire. Dietary data was grouped into 13 food groups. Alterations in food group intakes were categorized in tertiles; middle tertile of intake changes was considered as the reference category and the first and last tertiles of changes as increased and decreased intakes, respectively. Weight change per year of follow-up was the outcome of interest. Weight gain was defined as ≥ 0.5 kg/y, weight loss as ≤ -0.5 kg/y and stable weight as > -0.5 to < 0.5 kg/y. Multinomial logistic regression was used with stable weight as the reference group.

**Results:**

In men, weight loss was significantly predicted only by decreased intake of added sugars (OR: 2.21, 95% CI: 1.06-4.63). In women, weight gain was significantly predicted by decreased intake of whole grains (OR: 1.92, 95% CI: 1.11-3.31) and weight loss was predicted by decreased intake of vegetables (OR: 0.44, 95% CI: 0.21-0.91).

**Conclusions:**

Alterations in consumption of whole grains, vegetables, and added sugars are associated with body weight change, suggesting that it could be helpful in weight control.

## 1. Background

Obesity, a major public health problem worldwide, is associated with a significant burden of chronic diseases (1). The prevalence of obesity has increased globally during the past decades (2). Since losing weight is a big challenge, prevention of weight gain is a priority (3). However, weight gain often occurs gradually over a lifetime, due to imbalances between energy intake and expenditure leading to changes in body weight over time. Several lifestyle factors affect maintaining energy balance (3), of which dietary factors are the major modifiable ones. However, contribution of dietary factors to weight change has been difficult to clarify (3, 4).

Compared to single nutrient-based recommendations, food group-based recommendations could be more easily understood by people, because nutrients are consumed via foods. Furthermore, a systematic review showed that the proportion of macronutrients in diet was not important in predicting weight changes (4). Cross-sectional studies showed no consistent association between body weight and food group intake, and these studies could not identify which food group intakealterations are susceptible to body weight changes (5). Most studies of weight change over time evaluated baseline behaviors including food group intakes (4, 6-12). However, changes in dietary behaviors overtime may be more relevant. Only a few prospective studies have considered the effects of spontaneous changes in food group intakes over time on body weight changes (3, 13, 14). On the other hand, Kahn et al. considered only subjects with stable dietary behavior over 10 years and examined the associations between their dietary intakes and body mass index change and the likelihood of weight gain at the waist (15). Drapeau et al. reported that a self-reported decrease in consumption of food in the fat group or an increase in consumption of fruit group, predicted a lower increase in body weight (13). Koh-Banerjee et al. in a prospective cohort showed that an increase in whole-grain intake was inversely associated with long-term weight gain (14). Another study showed that weight change was associated with intake of potatoes, sugar-sweetened beverages, meats, fruits, vegetables, whole grains, nuts and yogurt (3). Most of these studies evaluated the effects of changes in food group intakes on weight gain and they did not include subjects who lost weight.

## 2. Objectives

The objective of this prospective study was to examine whether alterations in food group intakes over a 3 year follow-up period could be associated with subsequent weight changes. We also aimed to identify changes in food group intakes predictive of weight gain or weight loss in a cohort of Tehranian adults, with stable weight group as the reference.

## 3. Materials and Methods

### 3.1. Study Population

Subjects for this analysis were selected from the Tehran Lipid and Glucose Study (TLGS). Briefly, TLGS is a community-based prospective study conducted to investigate and prevent non-communicable diseases, in a representative sample of residents of district 13 of Tehran, the capital city of Iran. The first phase of the TLGS began in March 1999 and data collection is ongoing at three-year intervals (16, 17). For the current longitudinal study, of 3168 men and women aged 18 years and older, who attended the third phase of TLGS in 2005-2008 (baseline examination), 2640 subjects participated at the fourth phase of TLGS between 2008 and 2011 (follow-up examination). We excluded participants who were pregnant, those with cancer and stroke, and those who consumed drugs affecting body weight (corticosteroids, levothyroxine, and other hormonal medicines) at either baseline or follow-up (n = 2204). Furthermore, we excluded those who had no follow-up information on anthropometric measures or diet, those under- or over reported dietary intakes (the ratio of reported energy intake over estimated energy requirement which was not in ± 2 SD range (18, 19)), and those with extreme changes in annual weight (> 5 kg/y). Finally, the current analysis was data of 851 participants (378 men and 473 women), followed for a mean duration of approximately 3 years. Informed written consents were obtained from all participants and the study protocol was approved by the research council of the Research Institute for Endocrine Sciences (RIES), Shahid Beheshti University of Medical Sciences. The study was conducted according to the Declaration of Helsinki and was approved by the ethical committee of the Research Institute for Endocrine Sciences, Shahid Beheshti University of Medical Sciences. 

### 3.2. Assessment of Variables

#### 3.2.1. Body Weight

Body weight was measured at baseline and follow-up by trained technicians. Weight was measured to the nearest 100 g using digital scales, while the participants were minimally clothed, without shoes. Absolute body weight change was calculated by subtracting baseline weight from follow-up weight. Weight change per years of follow-up was calculated. Therefore, negative values indicated weight loss and positive values indicated weight gain. Annual weight change was classified as weight gain (≥ 0.5 kg/y), weight loss (≤ -0.5 kg/y), or stable weight (> -0.5 to < 0.5 kg/y). 

#### 3.2.2. Dietary Intake and Food Groups

Dietary data was collected using a validated semi-quantitative food frequency questionnaire (FFQ) developed for TLGS with 168 food items at baseline and follow-up. Trained dietitians asked participants about their intake frequency for each food item consumed during the past year on a daily, weekly, or monthly basis. The validity and reliability of the FFQ for food group intakes were assessed and were acceptable (20). Food items were summarized into 13 food groups including fruits, vegetables, whole grains, refined grains, potatoes, dairy foods, legumes, nuts, eggs, unprocessed red meats, processed meats, poultry and fish, and added sugars (Table 1). Absolute intakes of food groups were calculated in servings per day except for added sugars, the intake of which was calculated in percent of energy per day. Changes in intake of various food groups over 3 years were evaluated from differences between follow-up and baseline values. Alterations in food group intakes were categorized in tertiles.

**Table 1.** Definitions of Food Groups in the Study

Food Group	Foods in This Group
**Fruits**	all kinds of fresh and dried fruits, natural juices
**Vegetables**	all kinds of vegetable, mushrooms and herbage
**Whole Grains**	barbari bread, sangak bread, taftoon bread, whole-wheat bread and biscuits, cooked barley
**Refined Grains**	lavash and white-wheat bread, pasta, rice, macaroni, noodle, spaghetti, vermicelli, cakes, biscuits, cracker
**Potatoes**	boiled, mashed and roast potatoes, french fries
**Dairy foods**	milk and milk products (high and low fat), yoghurt, cheese, dough, ice cream
**Legumes**	lentil, beans, pea, broad bean, soya, split peas
**Nuts**	all kinds of nuts and seeds
**Eggs**	eggs
**Unprocessed Red Meats**	beef, lamb, hamburger, mutton, liver, brain, heart, tongue
**Processed Meats**	all kinds of sausages
**Poultry and Fish**	poultry, fish, canned fish
**Added Sugars**	Sugars and syrups added to foods or beverages when they are processed or prepared. This does not include naturally occurring sugars such as those in milk and fruits.

#### 3.2.3. Lifestyle Information

Trained interviewers collected baseline characteristics using a questionnaire. Information on age (years), educational level (with or without university degree), smoking behavior (yes/no), and physical activity level (MET-hr/wk) were also assessed at baseline.

Participants who smoked daily or occasionally were considered current smokers, and those who had never smoked or those who stopped smoking were considered non-smokers. Physical activity level was assessed using the Persian translated Modifiable Activity Questionnaire (MAQ) (21). High reliability and relatively moderate validity were reported for the Persian translated MAQ in Tehranian adults (22). The frequency and time spent on light, moderate, hard and very hard intensity activities according to the list of common activities of daily life over the past year were documented. Physical activity levels were expressed as metabolic equivalent hours per week (METs h/wk). 

### 3.3. Statistical Analysis

Differences in baseline characteristics between men and women were tested using Independent samples t-test, Mann-Whitney U test and chi-square test. Wilcoxon test was performed to examine the differences of food group intakes between baseline and follow-up in men and women. Differences in frequency distribution of weight changes between men and women were tested using chi-square test. We used General linear model analyses to appraise the interaction between sex and changes in food group intakes on subsequent weight changes. Interactions between changes in vegetables and eggs intakes and sex were statistically significant (P < 0.01). Therefore, stratified analyses were conducted by sex. Multinomial logistic regression analysis was used to assess the association between changes in food group intakes and the risk of weight gain or weight loss, compared to stable weight during follow-up. Odd ratio (OR) and 95% confidence interval (95% CI) were calculated with the middle tertile of intake changes as the reference category; the first tertile of changes in food group intakes was considered as increased intake and the last tertile of changes was considered as decreased intake of that food group. The covariates adjusted in the model were baseline age, body weight, university education, smoking behavior, and physical activity. Change in energy intake was not included as a covariate, because such a factor could be a mediator in the association between changes in food group intakes and body weight changes (in the casual pathway). All statistical analyses were conducted using SPSS (Version 15.0; SPSS Inc., Chicago, IL). P values < 0.05 were considered significant.

## 4. Results

Characteristics of participants are presented in Table 2. Age of participants ranged from 19 to 78 years. The mean change in body weight over time was 0.47 and 0.43 kg/y in men and women respectively and it was not significantly different between men and women. Significant differences between men and women were observed in baseline weight, physical activity, having university education and smoking behavior (P < 0.05). Mean intakes of several food groups differed significantly between baseline and follow-up in both sexes (Table 3). In men, intakes of vegetables and whole grains were increased, while intakes of refined grains, potatoes, legumes, eggs, unprocessed red meats, and added sugars were decreased during 3 years of follow-up (P < 0.01). In women, intakes of fruits, vegetables, whole grains, legumes, and nuts were increased; whereas, intakes of potatoes, unprocessed red meats, and added sugars were decreased during 3 years of follow-up (P < 0.05).

During the follow-up, we observed that 47.6% of men and 46.3% of women gained weight. Generally, 21.4% of men and 19.0% of women lost weight. The frequencies of subjects in different weight change categories did not differ between men and women (Table 4). Results of multinomial logistic regression analysis for the association of the alterations in food group intakes with the risk of weight gain or weight loss compared to stable weight as reference during follow-up are presented in Tables 5 and 6,men and women respectively. Regression models were adjusted for baseline age, body weight, university education, smoking behavior, and physical activity. 

In men, weight loss was significantly predicted only by decreased intake of added sugars. Decreased intake of added sugars compared to no change in added sugars intake, increased the likelihood of weight loss by 2.21 times (OR: 2.21, 95% CI: 1.06-4.63). In women, weight gain was significantly predicted by decreased intake of whole grains. After adjustment for potential confounding variables, we observed a 92% increase in the risk of weight gain for those women who decreased their intake of whole grains compared to the stable intake (OR: 1.92, 95% CI: 1.11-3.31). Moreover, weight loss was significantly predicted by decreased intake of vegetables in women. Decreased intake of vegetables compared to no change in vegetables intake, decreased the likelihood of weight loss in women by 56% (OR: 0.44, 95% CI: 0.21-0.91). In women, a decreased intake of eggs increased the likelihood of both weight gain and weight loss compared to stable weight. For the remaining food groups we found weak or no association with weight change.

**Table 2.** Characteristics of the Study Population: Tehran Lipid and Glucose Study ^a,b,c^

Variable	Men (n = 378)	Women (n = 473)	P Value
**Age, y**	40.2 ± 13.5	38.6 ± 11.7	0.067
**Baseline Weight, kg**	78.5 ± 12.7	66.1 ± 12.5	< 0.001
**Follow-up Weight, kg**	79.9 ± 12.9	67.4 ± 12.5	< 0.001
**Weight Change, kg/y**	0.47 ± 1.23	0.43 ± 1.18	0.70
**Physical Activity-MET, hr/wk**	53.5 ± 73.5	36.1 ± 43.3	< 0.001
**University Education**			
Yes	139 (36.9)	136 (29.2)	0.018
No	238 (63.1)	330 (70.8)	
**Smoking Behavior**			
Non-Smoker	302 (79.9)	463 (98.1)	< 0.001
Current Smoker	76 (20.1)	9 (1.9)	

^a^ Abbreviation: MET, metabolic equivalent.

^b^ Data are presented as means ± SD or No. (%).

^c^ P value for the characteristics differences between men and women tested with independent samples t-test, Mann-Whitney U test and chi-square test.

**Table 3.** Dietary Intakes of Participants at Baseline and After 3 Years: Tehran Lipid and Glucose Study ^a,b^

Dietary Intake (Servings/Day)	Men (n = 378)			Women (n = 473)		
	Baseline	Follow-up	P Value	Baseline	Follow-up	P Value
**Fruits**	2.3 ± 1.6	2.4 ± 1.9	0.69	2.4 ± 1.8	2.9 ± 2.4	< 0.001
**Vegetables**	2.1 ± 1.1	2.3 ± 1.2	0.001	2.4 ± 1.4	2.7 ± 1.4	< 0.001
**Whole Grains**	3.8 ± 3.3	6.3 ± 4.9	< 0.001	2.7 ± 2.8	4.5 ± 4.2	< 0.001
**Refined Grains**	6.6 ± 4.0	5.8 ± 3.8	0.002	4.9 ± 3.1	4.6 ± 3.0	0.056
**Potatoes**	0.2 ± 0.2	0.1 ± 0.1	< 0.001	0.2 ± 0.2	0.1 ± 0.1	< 0.001
**Dairy Foods**	2.0 ± 1.1	1.9 ± 1.0	0.39	2.0 ± 1.0	1.9 ± 1.0	0.67
**Legumes**	0.4 ± 0.4	1.0 ± 0.8	< 0.001	0.3 ± 0.4	0.9 ± 0.8	< 0.001
**Nuts**	0.4 ± 0.6	0.4 ± 0.7	0.57	0.3 ± 0.4	0.4 ± 0.7	0.02
**Eggs**	0.3 ± 0.3	0.3 ± 0.2	0.003	0.3 ± 0.2	0.3 ± 0.2	0.86
**Unprocessed Red Meats**	0.9 ± 0.8	0.7 ± 0.6	< 0.001	0.8 ± 0.8	0.6 ± 0.5	< 0.001
**Processed Meats**	0.2 ± 0.3	0.2 ± 0.2	0.76	0.2 ± 0.2	0.2 ± 0.2	0.13
**Poultry and Fish**	1.6 ± 1.3	1.6 ± 1.4	0.56	1.2 ± 1.0	1.3 ± 1.1	0.056
**Added Sugars (% of energy)**	6.3 ± 3.8	5.4 ± 3.1	0.001	5.3 ± 3.2	4.5 ± 2.7	< 0.001

^a^ Data are presented as means ± SD.

^b^ P value for the differences of food groups intake between baseline and follow-up in men and women tested with Wilcoxon test.

**Table 4.** Frequency Distribution of Weight Changes of Participants After 3 Years of Follow-up: Tehran Lipid and Glucose Study^a,b,c^

Weight Change	Men	Women
**Weight stable (± 0.5 kg/y)**	117 (31.0)	164 (34.7)
**Weight gain (≥ +0.5 kg/y)**	180 (47.6)	219 (46.3)
**Weight loss (≤ -0.5 kg/y)**	81 (21.4)	90 (19.0)

^a^ Data are presented as No. (%).

^b^ Weight change was calculated as follow-up weight minus baseline weight divided by years of follow-up.

^c^ Chi-Square value: 1.56, P: 0.46.

**Table 5.** Association Between Changes in Food Groups Intake and Weight Change in Men in Multinomial Logistic Regression Analysis: Tehran Lipid and Glucose Study ^a,b,c,d^

Food Groups	Increased Intake, OR (95% CI)		Decreased Intake, OR (95% CI)	
	Weight Gain	Weight Loss	Weight Gain	Weight Loss
**Fruits**	0.59 (0.32-1.12)	0.92 (0.43-1.95)	0.84 (0.45-1.58)	0.72 (0.33-1.59)
**Vegetables**	1.48 (0.77-2.82)	1.16 (0.52-2.62)	1.37 (0.71-2.62)	1.88 (0.84-4.18)
**Whole Grains**	1.14 (0.62-2.12)	0.89 (0.42-1.89)	1.18 (0.64-2.17)	0.92 (0.43-1.96)
**Refined Grains **	0.59 (0.32-1.10)	0.71 (0.32-1.55)	0.63 (0.33-1.18)	0.86 (0.39-1.89)
**Potatoes**	0.75 (0.40-1.42)	1.46 (0.67-3.21)	1.16 (0.62-2.16)	1.29 (0.58-2.89)
**Dairy Foods**	1.65 (0.87-3.14)	1.85 (0.84-4.11)	0.95 (0.51-1.76)	1.19 (0.55-2.57)
**Legumes**	0.71 (0.37-1.35)	0.85 (0.40-1.83)	0.86 (0.45-1.62)	0.77 (0.35-1.70)
**Nuts**	0.83 (0.44-1.59)	0.44 (0.19-1.01)	0.71 (0.37-1.39)	0.69 (0.31-1.52)
**Eggs**	0.79 (0.42-1.48)	0.79 (0.35-1.80)	0.82 (0.43-1.56)	1.43 (0.66-3.09)
**Unprocessed Red Meats**	0.66 (0.35-1.23)	0.82 (0.38-1.75)	0.64 (0.34-1.21)	0.80 (0.36-1.78)
**Processed Meats**	1.00 (0.54-1.87)	0.96 (0.44-2.07)	1.21 (0.62-2.35)	0.94 (0.42-2.09)
**Poultry and Fish**	1.40 (0.73-2.68)	1.14 (0.51-2.52)	1.15 (0.61-2.16)	0.74 (0.34-1.65)
**Added Sugars **	1.42 (0.79-2.52)	1.91 (0.90-4.03)	1.16 (0.65-2.09)	2.21 (1.06-4.63) ^e^

^a^ Abbreviations: CI, confidence interval; OR, odds ratio.

^b^ Regression models were adjusted for baseline age, body weight, university education, smoking behavior, physical activity and all the food groups in the table simultaneously.

^c^ Increased intake was defined as an increase in the number of servings per day for all items except added sugars (an increase in the percent of energy). Decreased intake was defined as a decrease in the number of servings per day for all items except added sugars (a decrease in the percent of energy). Increased and decreased intakes of food groups were compared to no change in intake (stable).

^d^ Ranges of tertiles of annual changes in food groups intakes (svg/d) were ≤ -0.59, -0.58-0.53, > 0.53 for fruits, ≤ -0.17, -0.16-0.55, > 0.55 for vegetables, ≤ 0.76, 0.77-3.80, > 3.80 for whole grains, ≤ -1.66, -1.65-0.62, > 0.62 for refined grains, ≤ -0.08, -0.07-0.01, > 0.01 for potatoes, ≤ -0.41, -0.40-0.36, > 0.36 for dairy foods, ≤ 0.31, 0.32-0.75, > 0.75 for legumes, ≤ -0.10, -0.09-0.09, > 0.09 for nuts, ≤ -0.03, -0.02-0.14, > 0.14 for eggs, ≤ -0.33, -0.32-0.05, > 0.05 for unprocessed red meats, ≤ -0.04, -0.03-0.05, > 0.05 for processed meats, ≤ -0.33, -0.32-0.41, > 0.41 for poultry and fish, and ≤ -1.96, -1.95-0.89, > 0.89 for added sugars (%/d).

^e^ Significant for comparison with the stable weight group.

**Table 6.** Association Between Changes in Food Groups Intake and Weight Change in Women in Multinomial Logistic Regression Analysis: Tehran Lipid and Glucose Study ^a,b,c,d^

Food Groups	Increased Intake, OR (95% CI)		Decreased Intake, OR (95% CI)	
	Weight Gain	Weight Loss	Weight Gain	Weight Loss
**Fruits**	1.31 (0.76-2.28)	1.19 (0.59-2.36)	1.03 (0.60-1.76)	0.73 (0.36-1.47)
**Vegetables**	0.84 (0.48-1.49)	0.78 (0.39-1.54)	0.90 (0.52-1.57)	0.44 (0.21-0.91) ^e^
**Whole grains**	1.67 (0.97-2.86)	1.03 (0.52-2.05)	1.92 (1.11-3.31)^e^	1.61 (0.81-3.19)
**Refined grains **	0.92 (0.54-1.58)	0.75 (0.37-1.53)	0.91 (0.52-1.58)	1.21 (0.61-2.39)
**Potatoes**	1.25 (0.72-2.17)	1.01 (0.51-2.00)	0.87 (0.49-1.52)	0.53 (0.26-1.11)
**Dairy foods**	1.27 (0.73-2.20)	1.58 (0.80-3.11)	1.07 (0.62-1.83)	1.11 (0.55-2.26)
**Legumes**	0.82 (0.48-1.42)	0.74 (0.38-1.48)	0.78 (0.46-1.34)	0.68 (0.34-1.34)
**Nuts**	0.68 (0.39-1.18)	0.69 (0.35-1.36)	0.69 (0.40-1.20)	0.67 (0.34-1.34)
**Eggs**	1.42 (0.82-2.45)	1.45 (0.72-2.93)	1.79 (1.02-3.15)^e^	2.08 (1.01-4.26) ^e^
**Unprocessed red meats**	1.13 (0.65-1.97)	0.81 (0.40-1.66)	1.00 (0.58-1.73)	0.80 (0.41-1.59)
**Processed meats**	1.17 (0.67-2.04)	0.92 (0.45-1.87)	1.17 (0.67-2.04)	1.05 (0.53-2.10)
**Poultry and fish**	0.99 (0.57-1.71)	1.47 (0.74-2.91)	0.96 (0.55-1.66)	0.96 (0.47-1.95)
**Added sugars **	1.45 (0.84-2.52)	1.46 (0.71-3.02)	1.34 (0.78-2.32)	1.45 (0.73-2.91)

^a^ Abbreviations: CI, confidence interval; OR, odds ratio.

^b^ Regression models were adjusted for baseline age, body weight, university education, smoking behavior, physical activity and all the food groups in the table simultaneously.

^c^ Increased intake was defined as an increase in the number of servings per day for all items except added sugars (an increase in the percentage of energy). Decreased intake was defined as a decrease in the number of servings per day for all items except added sugars (a decrease in the percentage of energy). Increased and decreased intakes of food groups were compared to no change in intake (stable).

^d^ Ranges of tertiles of annual changes in food groups intakes (svg/d) were ≤ -0.33, -0.32-0.98, > 0.98 for fruits, ≤ -0.33, -0.32-0.72, > 0.72 for vegetables, ≤ 0.12, 0.13-2.50, > 2.50 for whole grains, ≤ -0.93, -0.92-0.53, > 0.53 for refined grains, ≤ -0.09, -0.08-0.01, > 0.01 for potatoes, ≤ -0.42, -0.41-0.34, > 0.34 for dairy foods, ≤ 0.24, 0.25-0.64, > 0.64 for legumes, ≤ -0.04, -0.03-0.09, > 0.09 for nuts, ≤ -0.07, -0.06-0.07, > 0.07 for eggs, ≤ -0.27, -0.26-0.09, > 0.09 for unprocessed red meats, ≤ -0.02, -0.01-0.04, > 0.04 for processed meats, ≤ -0.21, -0.20-0.37, > 0.37 for poultry and fish, and ≤ -1.68, -1.67-0.61, > 0.61 for added sugars (%/d).

^e^ Significant for comparison with the stable weight group.

## 5. Discussion

The results of the present study showed that alterations in intake of specific food groups were associated with weight changes. In general, changes in intake of added sugars, vegetables and whole grains were predictive of weight change in Tehranian adults. In findings of previous studies concerning the effects of food group intake on weight, changes were not consistent. Some studies failed to show effects of intake of food items or groups on weight change, and others revealed different results. A cohort from two southern New England communities showed that none of the examined food groups, except for artificial sweeteners, were significantly related to weight change over a 4-year period (7). Fogelholm et al. in a systematic literature review which included 119 prospective cohort, case-control and interventional studies examined the role of foods in predicting weight change. They found probable evidence for high intakes of dietary fiber and nuts in predicting less weight gain and for high intakes of meat in predicting more weight gain. Suggestive evidence was found for a protective role of whole grains and dairy products against increasing weight. Moreover, high intakes of refined grains, sweets and desserts predicted more weight gain (4). These different effects in participants of various studies could be attributed to other life style or genetic factors.

The results of our study are in accordance with some studies, which observed a clear gender difference in associations of food group intakes with weight changes over time (8, 23). Schulz et al. showed that high fat food groups were strongly related to weight change in women, whereas in men, sweets intake was the only strong predictor of weight gain (8). Moreover, French et al. reported that increased consumption of French fries, dairy products, sweets and meat over a 2-year period predicted increases in weight in women, whereas in men, increased consumption of sweets and eggs was positively related to weight gain (23). In the present study, decreased intake of added sugars increased the likelihood of weight loss in men. This observation is similar to the findings from previous studies which showed that consumption of sweets and sugar-sweetened soft drinks was positively associated with weight gain (3, 6-8, 10, 23). Bes-Rastrollo et al. in a Mediterranean cohort observed that increased consumption of sugar-sweetened soft drinks, hamburgers and sausages were associated was higher risk of subsequent weight gain (6). A longitudinal study conducted in the United States reported that sugar-sweetened soft drinks were associated with increased weight gain in adult women (10). Moreover, Te Morenga et al. in a systematic review and meta-analyses of randomized controlled trials and cohort studies concluded that intake of free sugars or sugar sweetened beverages is a determinant of body weight (24).

In addition, we found that decreased intake of whole grains increased the likelihood of weight gain in women. This finding is in agreement with some studies reporting that increased consumption of whole grains was inversely related to weight gain (3, 14, 25). Koh-Banerjee et al. observed that increased consumption of whole grains was inversely associated with weight gain after adjusting for added bran or fiber; the dose-response relation appeared strongest for foods with ≥25% bran content by weight, and cereal fiber significantly protected against weight gain independent of fruit and vegetable fibers (14). Furthermore, Liu et al. reported that women in the highest quintile of change in whole-grain intake gained 1.07 kg over two 4-year intervals, whereas women in the lowest quintile gained 1.58 kg (25). Effects of whole grains on body weight may be partially mediated by fiber and constituents naturally found in whole grains including vitamins, minerals, phenolic compounds and phytoestrogens (26). In addition, properties of whole grains as compared with refined grains include lower glycemic index, greater particle size, and fewer calories because of their high fiber and water contents (27, 28). The results of the present study supported the evidence suggesting that high intakes of vegetables were related to lower weight gain (3, 9, 15, 29, 30). However, some studies reported no association between vegetable intake and weight change (7, 11). Vergnaud et al. in a large prospective study showed that higher baseline fruit and vegetable intakes, while maintaining constant total energy intakes, did not influence weight change substantially but could help reduce the risk of weight gain in persons who quit smoking (11). Conflicting results about the effect of vegetable consumption on body weight could be explained by differences in methods, populations, statistical analysis, follow-up periods, and the contexts in which individuals increased their consumption of vegetables. It is largely assumed that increased consumption of vegetables could displace more energy-dense foods from the diet, resulting in weight loss. Moreover, vegetables contain dietary fiber and flavonoids with anti-obesity effects (31, 32). Evidence suggests that fiber may exert beneficial role on preventing weight gain through several mechanisms, which include effects on satiety, glucose metabolism, energy density, absorption of macronutrients, secretion of gut hormones, the rates of ingestion and gastric emptying (33, 34). In this study, decreased intake of eggs increased the likelihood of both weight gain and weight loss compared to stable weight in women. In general, inverse association between intakes of any foods and weight change could be attributed to replacing other foods with higher energy density with them. However, inconsistent effect of eggs on weight change may be explained by various methods of cooking and preparing this food. In most of previous studies, dietary assessment was performed at baseline and information on changes of dietary habits during follow-up was not included. To compensate this limitation, at least in part, we assessed dietary intakes of participants twice, at baseline and again at the end of the study,then we evaluated changes in intake of various food groups over 3 years from differences between follow-up and baseline values. Another strength of the present study was consideration of some potential confounding factors including baseline age, body weight, university education, smoking behavior, and physical activity. Smoking habits are known to influence body weight (35). Therefore, smoking status was adjusted as a covariate in this study.

Moreover, the prospective design of this study and using multinomial logistic regression analysis to explore the effect of alterations in food group intakes on body weight changes over time are perceived as strengths. This approach allowed us to examine the effect of changes in food group intake on weight change groups, weight gain and weight loss separately, with stable weight as the reference group. These studies could be helpful in developing dietary recommendations based on foods, which are more practical than dietary recommendations based on nutrients. Consequently, this information could be advantageous in prevention of weight gain over time. This study does have some limitations. Evaluation of changes in food group intakes was performed by FFQs at 2 time points only, without considering intermediate changes in food group intakes. Moreover, weight change could lead to changes in dietary habit rather than vice versa. For example, persons who were gaining weight might have changed their food choices, which could have resulted in bias with respect to the observed associations. Furthermore, participants of longitudinal studies were volunteers and they could be a group of health-conscious individuals. Therefore, we have to be conscious in extrapolating our results to the general population. Finally, finding no associations between changes in some food group intakes and weight changes is not surprising, considering the FFQ limitations and the multifactorial aspects of body weight change. It is noticeable that the present study does not provide casual links between food group modifications and body weight changes.

Eating more or less of any foods would change the total amount of energy intake. However, the amount of associated weight change varies for specific foods. Differences in weight change seen for specific food groups could be due to various serving sizes, effects on satiety, displacement of other foods, and eating patterns. These results highlight the effect of dietary quality and food choices on weight control. In conclusion, our results showed that alterations in consumption of whole grains, vegetables, and added sugars are associated with body weight change during 3 years of follow-up in Tehranian adults. These findings maybe helpful in weight maintenance and weight control programs.
